# Complex Cystic Duct is Associated With Cholelithiasis

**DOI:** 10.1155/1998/25781

**Published:** 1998-08

**Authors:** Georgi P. Deenitchin, Junichi Yoshida, Kazuo Chijiiwa, Masao Tanaka

**Affiliations:** 1 Department of Surgery 1 Kyushu University Faculty of Medicine Fukuoka 812-82 Japan; 2 Department of Surgery Shimonoseki City Hospital 1-13-1 Koyo-cho Shimonoseki 750 Japan

## Abstract

The relationship between complex cystic ducts and cholelithiasis has seldom been investigated quantitatively. Thus we attempted a retrospective survey on two case series with and without cholelithiasis in a university hospital. A total of 500 patients who underwent endoscopic retrograde cholangiography were reviewed, 250 of whom had cholelithiasis and another 250 no gallstones. They were sampled at random during the period from 1979 through 1993. Parameters including the length, inner diameter and configuration of the cystic duct, and the angle formed by the cystic duct, and the axis of the gallbladder were compared between the groups with or without cholelithiasis. The patients with gallstones has significantly (*p*<0.001) longer and narrower cystic ducts (a mean of 48mm and 4mm in length and diameter, respectively) than did those without stones (a mean of 28mm and 7mm, respectively). Moreover, patients with gallstones showed a significantly (*p*<0.001) more acute angle between the gallbladder and the cystic duct than those without (a mean angle of 84° and 119° respectively). The overall frequency of the disfigurements of the cystic duct was significantly higher in the group with gallstones (99%) than in the group without (29%). The results therefore suggested that complex cystic ducts are associated with cholelithiasis.


*HPB Surgery*, 1998, Vol. 11, pp. 33-37
Reprints available directly from the publisher
Photocopying permitted by license only

© 1998 OPA (Overseas Publishers Association) N.V.
Published by license under
the Harwood Academic Publishers imprint,
part of The Gordon and Breach Publishing Group.
Printed in India.

### Complex Cystic Duct is Associated with Cholelithiasis

GEORGI P. DEENITCHIN*, JUNICHI YOSHIDA**, KAZUO CHIJIIWA and MASAO TANAKA

*Department of Surgery 1, Kyushu University Faculty of Medicine, Fukuoka 812-82, Japan*

*(Received 6 January 1997; In final form 30 April 1997)*

**The relationship between complex cystic ducts and cholelithiasis has seldom been investigated quantitatively. Thus we attempted a retrospective survey on two case series with and without cholelithiasis in a university hospital. A total of 500 patients who underwent endoscopic retrograde cholangiography were reviewed, 250 of whom had cholelithiasis and another 250 no gallstones. They were sampled at random during the period from 1979 through 1993. Parameters including the length, inner diameter and configuration of the cystic duct, and the angle formed by the cystic duct, and the axis of the gallbladder were compared between the groups with or without cholelithiasis. The patients with gallstones has significantly ($p < 0.001$) longer and narrower cystic ducts (a mean of 48 mm and 4 mm in length and diameter, respectively) than did those without stones (a mean of 28 mm and 7 mm, respectively). Moreover, patients with gallstones showed a significantly ($p < 0.001$) more acute angle between the gallbladder and the cystic duct than those without (a mean angle of 84° and 119° respectively). The overall frequency of the disfigurements of the cystic duct was significantly higher in the group with gallstones (99%) than in the group without (29%). The results therefore suggested that complex cystic ducts are associated with cholelithiasis.**

*Keywords:* Cystic duct, cholelithiasis

### INTRODUCTION

Abdominal pain in some patients has been ascribed to a variety of complexity of the cystic duct. They include the narrowing, kinking, tortuosity and sharp angulation of the cystic duct relative to the axis of the gallbladder or to the common bile duct, the flow in which may resist bile emptying from the gallbladder. Hence this entity has been named as the cystic duct syndrome [1-3]. On the other hand, bile stasis in the gallbladder has been considered as one of the important lithogenic factors [4-14]. An increase in the cystic duct resistance or its occlusion has been shown to form sludge and eventually stones in the gallbladder [6-8, 13, 15-17]. Brugge *et al.* [7] and Rizk and Deshmukh [18] linked the cystic duct syndrome with an early phase of gallstone formation.

To better clarify the causative effect of the cystic duct resistance in lithogenesis, we compared several anatomical parameters and the frequency of cystic duct abnormalities between two groups with and without cholelithiasis. The

*Dr. Deenitchin has been on leave of absence from Bulgaria.
**Address for correspondence: Department of Surgery, Shimonoseki City Hospital, 1-13-1 Koyo-cho, Shimonoseki 750, Japan.

parameters included the angle between the cystic duct and the axis of the gallbladder, because we considered that it reflected the direction onto which the force of the gallbladder contraction is applied.2

### SUBJECTS AND METHODS

### 1. Subjects

During the period 1979 through 1993, a total of 2,161 patients underwent endoscopic retrograde cholangiography (ERC). Of them, 500 patients were selected at random; 250 of them had gallbladder stones and the remaining 250 no calculi. All the patients met a criterion of visualizing the whole length of the cystic duct. Another criterion of exclusion was to rule out cases with concomitant diseases that may affect the course of the cystic duct, such as tumor of the pancreatic head, stenosis of the papilla, carcinoma of the biliary tract and gallstones impacted in the cystic duct.

The group of patients without gallstones underwent ERC with the suspicion of various pancreatic and biliary tract diseases. Sixty of these patients had polyps in the gallbladder, 39 hepatolithiasis, 28 chronic pancreatitis, 24 liver cirrhosis, and 12 pancreatic cysts. Patients with gallbladder polyps underwent ERC to detect neoplasms of the gallbladder. The remaining 87 patients showed no remarkable findings.

### 2. Methods

On ERC films, the length and inner diameter of the cystic duct were measured and corrected using the diameter of a duodenoscope (model JF-IT 20 or JF 100, Olympus Optical Co., Tokyo, Japan) as a reference. We measured the angle formed by the cystic duct and the axis of the gallbladder. Cystic duct abnormalities were defined as follows: a length of 40 mm or more, complex configurations of the gallbladder and the cystic duct, such as a coiled or tortuous cystic duct in a form of Σ or siphon (Fig. 1), the drainage of the cystic duct into the left side of the common bile duct or into the left hepatic duct, and a cystic duct with an acute angle against the common bile duct (Fig. 2) suggesting resistant emptying of the gallbladder bile.

In the statistical analysis, data are expressed as mean values ± standard deviations. Comparison of continuous variables between two groups was examined by two-tailed Student's

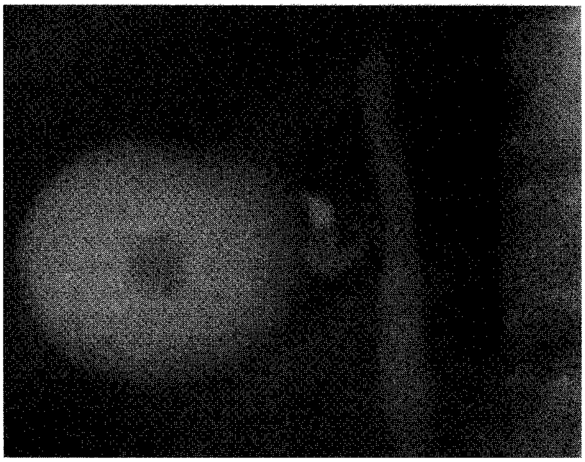

FIGURE 1 Endoscopic retrograde cholangiography showing the cystic duct in a "siphon" form.

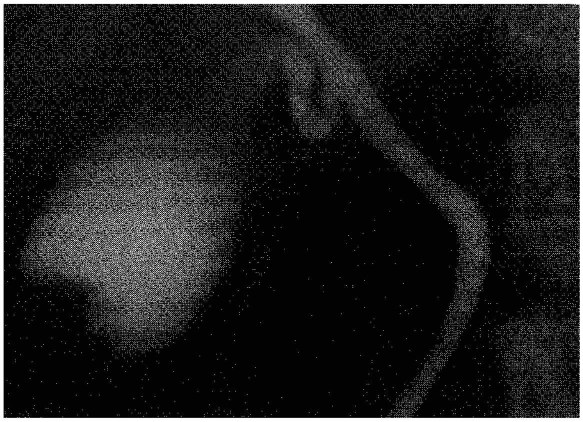

FIGURE 2 Endoscopic retrograde cholangiography demonstrating the cystic duct that joins the common bile duct against bile flow therein.

*t*-test whereas discontinuous variables by $\chi^2$ test. A *p*-value less than 0.05 was considered statistically significant.

### RESULTS

### 1. Demographic Data

The mean age of 250 patients with gallstones was 55.8 years and in 250 patients without calculi 55.5 years. The sex distribution was also comparable between the two groups, men accounting for 146 patients among those with stones (58.4%) and 127 among those without calculi (50.8%).

### 2. Quantitative Results

The length of the cystic duct in the groups with and without gallstones measured 47.8±17.5 mm and 27.8±8.9 mm, respectively: the group having stones showed a significantly longer cystic duct ($p < 0.001$). The inner diameter of the cystic duct in the groups with and without calculi was 4.1±2.4 mm and 6.5±2.7 mm, respectively. Thus patients with gallstones had significantly narrower cystic duct ($p < 0.001$).

In the calculous and acalculous groups, the angle between the cystic duct and the axis of the gallbladder showed a mean of 84.0±40.3° (sharp angle) and 118.8±32.2° (wide angle), respectively. Thus the group with stones had a significantly more acute angle than did the group without ($p < 0.001$).

### 3. Qualitative Results

Various disfigurements of the cystic duct are listed in Table I. The overall frequency of having one or more of these abnormalities was significantly higher in the group with gallstones (99% or 248/250) than in those without (29% or 73/250) ($p < 0.001$).

A combination of two abnormalities was noted in 128 patients of the group with gallstones (51.2%) while in 11 patients (4.4%) of the acalculous group, the difference being significant ($p < 0.001$). In the group with stones, 49 patients (19.6%) had three abnormalities in combination and seven (2.8%) had four in combination (Tab. II).

Gallbladder polyps coexisted in 26 patients of the group with stones (10.4%). In the group without calculi, 60 patients (24.0%) had polyps of the gallbladder, 30 of which (50.0%) had a disfigured cystic duct; in the remaining 190 patients having no polyps, the cystic duct was abnormal in 43 (22.6%). Thus in the acalculous group, the frequency of complex cystic ducts in patients with polyps were twice as high as in those without polyps; the difference was significant ($p < 0.01$).

All the specimens after cholecystectomy were histologically examined. No abnormalities were noted in the cystic duct.

TABLE I Classification of the abnormalities of the cystic duct with only one abnormality in calculous group

| | Type of anomaly | Number of patients |
|---|---|---|
| 1. | Cystic duct, longer than 40 mm | 6 |
| 2. | Acutely angulated cystic duct | 8 |
| 3. | Acute angulation of infundibulum of the gallbladder | 13 |
| 4. | Narrowing of the cystic duct | 13 |
| 5. | Retrograde obstruction of the cystic duct | 13 |
| 6. | Tortuosity of the cystic duct | 7 |
| 7. | Coiled cystic duct | 3 |
| 8. | The cystic duct entering the CBD against the bile flow in the CBD | 1 |
| | Total (%) | 64 (25.6%) |

CBD – common bile duct.

TABLE II Patients having one or more abnormalities of the cystic duct

| Number of abnormalities | Group: With stones | Without stones | P |
|---|---|---|---|
| 1 | 64 (25.6%) | 62 (24.8%) | NS |
| 2 | 128 (51.2%) | 11 (4.4%) | <0.001 |
| 3 | 49 (19.6%) | – | – |
| 4 | 7 (2.8%) | – | – |
| 1 or More | 248 (99.2%) | 73 (29.2%) | <0.001 |

NS – not significant.

### DISCUSSION

The present study demonstrated that a variety of abnormalities were more frequent in patients having cholelithiasis than in those without. Clear delineation of the cystic duct on ERC allowed us a detailed analysis on the various anatomic parameters of the cystic duct likely to cause stasis of the gallbladder bile. Quantitative analysis of the cystic duct anatomy is novel to our knowledge.

Previous investigators have revealed that gallbladder stasis predisposes to formation of cholesterol stones from supersaturated bile [4, 6, 10]. Some reports focused on the importance of cystic duct abnormalities in gallbladder lithogenesis. For example, an increase in the cystic duct resistance was claimed to be one of the etiologic factors for gallstone formation [6]. Camishion and Goldstein [1] described a spectrum of cystic duct anomalies among patients with the cystic duct syndrome, listing partial obstruction, narrowing, kinking, tortuosity, the acute angulation of the gallbladder infundibulum and the cystic duct, and drainage of the cystic duct into one of the hepatic ducts. Gowen [3] reported similar results in on investigation using ERC films in patients with the cystic duct syndrome. These authors suggested that emptying of the gallbladder was impaired because of organic obstacles in the cystic duct.

On lithogenesis in the gallbladder, Rizk and Deshmukh [18] and Brugge *et al.* [7] suggested a causative effect of the cystic duct complexity. Eberle and Rettenmaier [19] and Wolpers and Hoffman [13] also reported that complete or partial obstruction of the cystic duct lead to formation of sludge and subsequent lithogenesis.

Our results support the idea that gallstone formation is facilitated by impaired emptying of the gallbladder because of frequently multiple disfigurements in the cystic duct. The group with gallstones had a longer or narrower cystic duct than those without calculi. Because the resistance of a tube is in general inversely proportional to the fourth power of its radius, a small change in the caliber of the cystic duct will have an exponential effect on the flow through this duct [6].

Moreover, the angle formed by the axis of the gallbladder and the cystic duct was more acute in the group with stones (84°) than in the group without (119°). This difference may have partially contributed to lithogenesis seen in the group with calculi. Several anatomic factors of lithogenesis frequently coexisted in a patient, thus providing a milieu within the gallbladder prone to the formation of gallstones. In addition, gallbladder polyps were noted more frequently among patients with disfigured cystic ducts in the absence of cholelithiasis. In the adult Japanese population undergoing echography for screening, the incidence of gallbladder polyps are 10% in the male and 7% in the female (unpublished data). The incidence of 24% in the acalculous group in our series was higher than those, because selected patients underwent ERC on account of abnormalities of elevated lesions in the gallbladder.

Whether the anatomic abnormality is of inborn or acquired nature is difficult to discern. Moreover narrowing of the cystic duct may lead to calculi formation but may have been caused by fibrosis after passage of gallstones. Initial events unknown, gallbladders harboring multiple small stones are thus associated with narrow cystic ducts, letting some stones out and seeing the outlet narrowing and bile stasis within.

In conclusion, factors with a possible influence on the gallbladder emptying such as the length, width, angulation and specific anomalies of the cystic duct provide a background for lithogenesis. The relation between the cystic duct configuration and the gallbladder emptying, however, remains to be investigated.

### *References*

[1] Camishion, R. C. and Goldstein, F. (1967). Partial, noncalculous cystic duct obstruction (cystic duct syndrome). *Surgical Clinics of North America*, **47**, 1107–1114.

[2] Halverson, J. D., Garner, B. A., Siegel, B. A., Alexander, R., Edmundowicz, S. A., Campbell, W. and Miller, J. E. (1992). The use of hepatobiliary scintigraphy in patients with acalculous biliary colic. *Archives of Internal Medicine*, **152**, 1305–1307.

[3] Gowen, G. F. (1984). Endoscopic retrograde cholangiopancreatography in the diagnosis of cystic duct syndrome. *Surgery, Gynecology and Obstetrics*, **159**, 217–221.

[4] Thureborn, E. (1965). Formation of gallstone in man. *Archives of Surgery*, **91**, 952–957.

[5] Pomeranz, I. S. and Shaffer, E. A. (1985). Abnormal gallbladder emptying in a subgroup of patients with gallstones. *Gastroenterology*, **88**, 787–791.

[6] Pitt, H. A., Doty, J. E., DenBesten, L. and Kuchenbecker, S. L. (1982). Stasis before gallstone formation: Altered gallbladder compliance or cystic duct resistance? *American Journal of Surgery*, **143**, 144–149.

[7] Brugge, W. R., Brand, D. L., Atkins, H. L., Lane, B. P. and Abel, W. G. (1986). Gallbladder dyskinesia in chronic acalculous cholecystitis. *Digestive Diseases and Sciences*, **31**, 461–468.

[8] Soloway, R. D., Trotman, B. W. and Ostrow, J. D. (1977). Pigment gallstones. *Gastroenterology*, **72**, 167–182.

[9] Paumgartner, G. and Sauerbruch, T. (1991). Gallstones: pathogenesis. *Lancet*, **338**, 1117–1121.

[10] Rains, A. J. H. (1962). Researches concerning the formation of gallstones. *British Medical Journal*, **2**, 685–691.

[11] LaMorte, W. W., Schoetz, D. J. Jr., Birkett, D. H. and Williams, L. F. Jr. (1979). The role of the gallbladder in the pathogenesis of cholesterol gallstones. *Gastroenterology*, **77**, 580–592.

[12] Fiorucci, S., Scionti, L., Bosso, R., Desando, A., Bottini, P., Marino, C. and Morelli, A. (1992). Effect of erythromycin on gallbladder emptying in diabetic patient with and without autonomic neuropathy and high levels of motilin. *Digestive Diseases and Sciences*, **37**, 1671–1677.

[13] Wolpers, C. and Hofmann, A. F. (1993). Solitary versus multiple cholesterol gallbladder stones. Mechanism of formation and growth. *Clinical Investigation*, **71**, 423–434.

[14] Tierney, S., Pitt, H. A. and Lillemoe, K. D. (1993). Physiology and pathophysiology of gallbladder motility. *Surgical Clinics of North America*, **73**, 1267–1290.

[15] Bennett, F. D., DeRidder, P., Koloszi, W., Gordon, R. and Rapp, J. (1985). Cholecystokinin cholescintigraphic findings in the cystic duct syndrome. *Journal of Nuclear Medicine*, **10**, 1123–1108.

[16] Wolpers, C. (1986). Changes in silent gallbladder stones. Roentgen-diagnostic and symptomatologic observations over 30 years. *Deutsche Medizinische Wochenschrift*, **111**, 1186–1191.

[17] Grossman, S. J. and Joyce, J. M. (1991). Hepatobiliary imaging. *Emergency Medicine Clinics of North America*, **4**, 853–874.

[18] Rizk, T. A. and Deshmukh, N. (1993). Familial acalculous gallbladder disease. *Southern Medical Journal*, **86**, 183–186.

[19] Eberle, F. and Rettenmaier, G. (1984). Gallbladder sludge: a sonographically recognizable stage of lithogenesis. *Zeitschrift der Gastroenterologie*, **2**, 82–87.

